# Editorial: Human inspired robotic intelligence and structure in demanding environments

**DOI:** 10.3389/fnbot.2022.1026917

**Published:** 2022-09-14

**Authors:** Jiahao Chen, Hang Su, Juan Sandoval, Longbin Zhang, Shanlin Zhong

**Affiliations:** ^1^Institute of Automation, Chinese Academy of Sciences, Beijing, China; ^2^Politecnico di Milano, Milan, Italy; ^3^HSE Department, University of Poitiers, Poitiers, France; ^4^Department of Engineering Mechanics, KTH Royal Institute of Technology, Stockholm, Sweden

**Keywords:** demanding environments, human–robot interaction, robotic intelligence, dexterous manipulation, sensorimotor coordination

With the development of robotic research, robots play an increasingly important role in academic research and industry. However, the application and development of robots in a demanding environment with limited information and much unpredictability such as high-precision assembly, surgical operation, operation in deep space or ocean, rehabilitation, remote service, and remains hampered. Humans have an innate ability to perform these tasks with relative ease due to the integration and coordination of perception, decision, motion control, and the musculoskeletal system, even in demanding environments. Establishing human-inspired robotic intelligence and structures from the imitation of neural mechanisms to body structures may be a potential way to improve the performance of robots in demanding environments.

Based on the above observation, this Research Topic hopes to discuss how to select key mechanisms related to human performance and promote human-inspired robotic intelligence and structure with these neural mechanisms. This Research Topic will present recent advanced algorithms and applications within human-inspired robotic intelligence and structure in demanding environments, thereby providing a comprehensive overview for future directions in both theoretical and engineering aspects. Authors are encouraged to submit original research and review papers that present state-of-the-art research and applications of human-inspired robotic intelligence and structure in demanding environments.

This Research Topic contains 7 research articles, where five related to robotic perception, decision, and control in complex environments and tasks, and two related to the skill learning of human–robot interaction.

To meet the increasing requirements of robotic intelligence in demanding environments and task, the perception, decision, and control of robots should be improved and integrated. For the improvement of perception in changing environments and tasks, Liang et al. proposed a new incremental learning framework for domain-incremental cases to continually make the model adapt from one domain to another at multiple levels. Inspired by the characteristics of the human brain in progressive learning and continuous learning, it can significantly avoid catastrophic forgetting, mitigate performance degradation in the previous domains, and improve the object detection accuracy of the novel target domain.

Furthermore, the decision of robot is also critical and required to explore unknown environments, especially in hazardous conditions unsuitable for humans. A distributed small-step path planning method using modified reinforcement learning is proposed by Tang et al. Based on this method, robots can inspect and evaluate post-earthquake building damage in complex and unstable environments with unexpected obstacles. Compared with traditional unplanned detection method, this method achieves higher accuracy. To realize robotic searching tasks in complex environments, Chen et al. proposed a training framework using supervised learning and reinforced learning successively for a multi-continuous-output fuzzy inference system. The method is verified and demonstrates that the system trained by the proposed framework can achieve around a 10% higher success rate it can significantly avoid catastrophic forgetting, mitigate performance degradation in the previous domains, and improve the object detection accuracy of the novel target domain.

Besides, for the control of robots in demanding environments and tasks, some novel methods have been proposed in this Research Topic. To realize compliant industrial operations like grinding 3C products, Xue et al. proposed an adaptive compliant control framework for robotic interaction. This method plans reference trajectory with dynamic movement primitives and realizes compliant control with the combination of feedforward, impedance, and admittance controller. Based on this method, an Elite robot can grind a computer mouse compliantly with interaction force within a certain expected range. To realize intelligent and high-precision control of rock drilling robots with characteristics of heavy load, large span, and multi-joints, Zhou X. et al. proposed a hybrid positional error compensation method based on Radial Basis Function Network and Light Gradient Boosting Decision Tree to effectively compensate both geometric and non-geometric errors.

In addition, robots can enhance the efficiency and ability of human in some complex and repetitive tasks through human–robot interaction. In this Research Topic, to realize human-robot collaboration and synchronous operation with human in the same workspace and time, Zhou et al. proposed a cooperative shared control scheme based on intention recognition for flexible assembly manufacturing. With this method, a robot can effectively recognize human intention based on a three-dimensional point cloud, select suitable tools, avoid obstacles, and realize smooth cooperation and synchronous operation with human. To enable the elderly sit-to-stand (STS) comfortably, happily, and safely, Wang et al. proposed an STS movement analysis of the center of gravity method with the utilization of Vicon optical motion capture system and automatic moving target indicator 3-D force measuring table. Based on this method, a movement curve and related dynamic parameters that can genuinely reflect the STS situation of the elderly can be obtained, which is beneficial to the future design of relevant auxiliary standing devices and technologies.

## Author contributions

JC wrote the manuscript. HS, JS, LZ, and SZ helped to improve the manuscript. All authors contributed to the manuscript and approved the submitted version.

## Funding

This work was supported by the State Key Laboratory of Management and Control for Complex Systems under Grant E2S9020904.

## Conflict of interest

The authors declare that the research was conducted in the absence of any commercial or financial relationships that could be construed as a potential conflict of interest.

## Publisher's note

All claims expressed in this article are solely those of the authors and do not necessarily represent those of their affiliated organizations, or those of the publisher, the editors and the reviewers. Any product that may be evaluated in this article, or claim that may be made by its manufacturer, is not guaranteed or endorsed by the publisher.

